# Enhancement of A Cationic Surfactant by Capping Nanoparticles: Synthesis, Characterization and Multiple Applications

**DOI:** 10.3390/molecules25092007

**Published:** 2020-04-25

**Authors:** A. Labena, M. A. Hegazy, W. M. Kamel, Amr Elkelish, Wael N. Hozzein

**Affiliations:** 1 Processes Development Department, Egyptian Petroleum Research Institute (EPRI), Nasr City, Cairo 11727, Egypt; 2 Petrochemical Department, Egyptian Petroleum Research Institute (EPRI), Nasr City, Cairo 11727, Egypt; mohamedhegazy997@gmail.com (M.A.H.); walaakamel_80@yahoo.com (W.M.K.); 3 Botany Department, Faculty of Science, Suez Canal University, Ismailia 41522, Egypt; amr.elkelish@science.suez.edu.eg; 4 Bioproducts Research Chair, Zoology Department, College of Science, King Saud University, Riyadh 11451, Saudi Arabia; whozzein@ksu.edu.sa; 5 Botany and Microbiology Department, Faculty of Science, Beni-Suef University, Beni-Suef 62511, Egypt

**Keywords:** cationic surfactant, mild steel, biocidal activity, corrosion inhibitor, Slime-Forming Bacteria (SFB)

## Abstract

There is scarce information on cationic surfactants’ biocidal and corrosion inhbibition effects on Slime-Forming Bacteria (SFB) isolated from oil field formation water. Therefore, this work focused on the the synthesis of a cationic surfactant (CS) to increase its features by capping different metal nanoparticles (zinc, ZnNPs-C-CS; manganese, MnNPs-C-CS and tin, SnNPs-C-CS) and used them as biocides and corrosion inhibitors. The cationic surfactant was synthesized and characterized by Fourier-Transform Infrared (FTIR) and Nuclear Magnetic Resonance (NMR) spectroscopy. Afterwards, different nanoparticles were synthesized, characterized, and exploited to cap by the CS. The CS and the different nanoparticles capped by the CS were tested for their antimicrobial susceptibility against standard bacterial and yeast strains. The synthesized compounds were further evaluated as anti-biofilms agents against positively-developed bacterial biofilms. Moreover, the CS and the ZnNPs-C-CS, MnNPs-C-CS, and SnNPs-C-CS were assessed as potential biocides against SFB, particularly *Pseudomonas* sp. (isolated from contaminated formation water), and as corrosion inhibitors against cultivated salinity. The results revealed the great effect of the different CS-capped NPs as broad-spectrum antimicrobial and anti-biofilm agents at lower Minimum Inhibitory Concentrations (MICs), Minimum Bactericidal Concentrations (MBCs), Minimum Fungicidal Concentrations (MFCs) and Minimum Biofilm Inhibitory Concentrations (MBICs), and the activities were reported in order of SnNPs-C-CS > MnNPs-C-CS > ZnNPs-C-CS > CS. Furthermore, the ZnNPs-C-CS, MnNPs-C-CS, and SnNPs-C-CS demonstrated biocidal and corrosion inhibition effects against *Pseudomonas* sp. at a salinity of 3.5% NaCl, with metal corrosion inhibition efficiencies of 88.6, 94.0 and 96.9%, in comparison to a CS efficiency of 85.7%. In conclusion, the present work provides a newly synthesized cationic surfactant and has enhanced its antimicrobial and its metal corrosion inhibition effects by capping different nanoparticles, and it has been successfully applied against slime-forming bacteria at a salinity of 3.5% NaCl.

## 1. Introduction

Microbially influenced corrosion or bio-corrosion can be attributed to metal destruction initiated or accelerated by the microbial activities on metal surfaces, in the form of localized pit and crevice corrosion [1]. Bio-corrosion affects many industries, including shipping, dentistry, pulp and paper industries, oil and gas industries, sugar industries, and cooling water systems [2,3]. Microorganisms, especially bacteria, accelerate corrosion reactions via their attachment to the metallic materials in the form of biofilms (microbial communities attached to the metallic surface by their self-producing extracellular polymeric matrix), aggressive metabolites produced such as sulfides, acid or oxidant substances or via increasing cathodic reactions [4,5]. Bacterial biofilm formation can cause extremely dangerous problems [6,7] and has a role in the material biofouling, which induces bad impacts in industrial fields as biofilms can not be eradicated easily with normal biocides or antibiotics [8,9]. Such a bacterial community induces severe pitting or crevice corrosion by the formation of differential aeration cells on the metallic materials. Carbon steels are the commonly used metallic materials in such industries, due to their attractive mechanical characteristics and comparably lower cost despite less resistance to biocorrosion. The biocorrosion can be represented by many bacterial communities either under anaerobic or aerobic conditions, such as Sulfate-Reducing Bacteria (SRB), Iron-Oxidizing Bacteria (IOB), Manganese-Oxidizing Bacteria (MOB), Iron-Reducing Bacteria (IRB) and Slime-Forming Bacteria (SFB) [10,11,12]. *Pseudomonas* sp. is considered one of the SFB and a well-known bacterium that is harbored in marine environments in the form of biofilm layers. In the oil and gas industries, proper corrosion inhibitors and biocides are the most practical and successful approaches for biocorrosion mitigation on metal surfaces [13]. Therefore, there is a critical demand for the synthesis of a novel biocide that has efficiency in inhibiting biofilm development on different surfaces. Promptly, many synthesized surfactants have been extensively applied as corrosion inhibitors and biocides [14,15]. Improvement of the surfactants’ structures leads to an increase in their surface characteristics and improves the inhibition efficiency of metal corrosion and the biocidal effect [16].

Lately, many studies on pyrrolidinium-derivative surfactants have been recorded. One synthesized a pyrrolidinium surfactant with 1,4-dichloro-butyne pyrrolidine and alkylbromides [17]. Afterwards, a synthesized 1,1′-(butane-1,s-alkyl)bis(1-alkylpyrrolid-inium) bromide was provided by reaction of pyrrolidine with alkyl bromide and 1,4-dibromobutane [18]. Moreover, a 1,1′-(ethane-1,2-diyl bis(oxy)) bis(2-oxoethane-1,2-diyl) bis(1-dodecyl pyrrolidin-1-ium) dibromide was obtained by reacting 1-dodecyl-pyrrolidine with ethane-1,2-diyl bis(2-bromoacetate) [19]. The above synthesized surfactants showed that pyrrolidinium-derivative surfactants have greater surface activities with many applicable procedures [17,18,19]. Nonetheless, there are still limited structural changes in pyrrolidinium-synthesized surfactants using pyrrolidine as a raw material. This prompted our team to synthesize a new structural pyrrolidinium-derivative surfactant. Recently, nanomaterials have attracted much attention in this respect, which is attributed to many features, such as their nano-scale size; higher ratio of surface area to volume; lower toxicity; higher medium stability adsorption of surface plasmon; remarkable surface coverage; curvature; functionalization; potential antimicrobial properties; ease of transportation, attachment and migration with the biofilm matrix; and disruption of the biofilm [20,21,22]. The synthesized nanoparticles capped by the synthesized cationic surfactat are progressively applied as a forceful anti-biofilm strategy. Therefore, this work focused on the synthesis of a cationic surfactant (CS) and different nanoparticles of zinc, manganese and tin capped by the CS (ZnNPs-C-CS, MnNPs-C-CS, and SnNPs-C-CS) in order to evaluate their potentiality as broad antimicrobial agents, anti-biofilm agents and as biocides and corrosion inhibitors for environmentally isolated and enriched *Pseudomonas* sp. under high salinity conditions. Moreover, this work will provide a solution for the metal corrosive effect of slime-forming bacteria by combining two powers in a product: different metal nanoparticles capped by a newly synthesized cationic surfactant.

## 2. Results and Discussion

### 2.1. The Synthesized Cationic Surfactant (CS)

**Figure 1:**
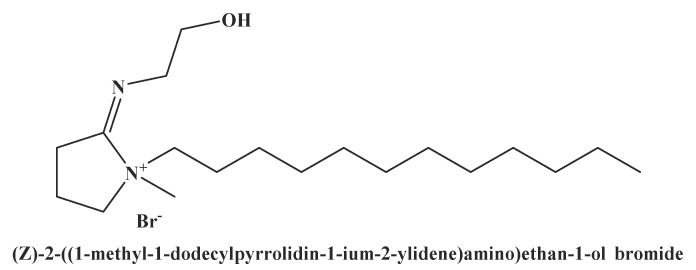
the structure of the synthesized cationic surfactant.

In the present work, the cationic surfactant, (*Z*)-2-((1-methyl-1-dodecyl pyrrolidin-1-ium-2-ylidene)amino)ethan-1-ol bromide, was successfully synthesized (see Figure 1)

### 2.2. Confirmation of the CS Structure

#### 2.2.1. FT-IR Spectroscopy

The Fourier-Transform Infrared Spectrum (FT-IR) spectrum of (*Z*)-2-((1-methyl-1-dodecyl pyrrolidin-1-ium-2-ylidene)amino)ethan-1-ol bromide displayed as follows: bands at 731.11 cm^−1^ ((CH_2_)n rocking), 1072.28 cm^−1^ (C-N+), 1378.54 cm^−1^ (CH_3_ symmetric bending), 1466.09 cm^−1^ (CH_2_ asymmetric bending), 1630.29 cm^−1^ (C=N), 2854.61 cm^−1^ (CH symmetric stretching), 2925.59 cm^−1^ (CH asymmetric stretching) and 3427.61 cm^−1^ (OH stretching). The FT-IR spectrum proved the predicted functional groups in the CS (Figure S1).

#### 2.2.2. H NMR Spectroscopy

^1^H NMR spectrum of (*Z*)-2-((1-methyl-1-dodecyl pyrrolidin-1-ium-2-ylidene)amino)ethan-1-ol bromide. The ^1^H NMR (DMSO) spectrum exhibited different bands at δ = 0.848 ppm (t,3H, NCH_2_(CH_2_)_9_CH_2_C**H**_3_); δ = 1.223 ppm (m, 18H, NCH_2_CH_2_(C**H**_2_)9CH_3_); δ = 1.862 ppm (m, 2H, NCH_2_C**H**_2_(CH_2_)_9_CH_3_); δ = 3.176 ppm (t, 2H, NC**H**_2_CH_2_(CH_2_)_9_CH_3_); δ = 2.405 ppm (s, 6H, NCCH_2_C**H**_2_CH_2_N); δ = 2.034 ppm (s, 6H, NCC**H**_2_CH_2_CH_2_N); δ = 3.014 ppm (s, 6H, NC**H**_2_CH_2_CH_2_N); δ = 2.834 ppm (s, 6H, NC**H**_3_); δ = 3.404 ppm (t, 2H, NC**H**_2_CH_2_OH); δ = 3.830 ppm (t, 2H, NCH_2_C**H**_2_OH); δ = 3.999 ppm (s, 1H, NCH_2_CH_2_O**H**). The data on ^1^HNMR spectra proved the distribution of hydrogen protons in the CS (Figure S2).

### 2.3. Structure Confirmation of the Prepared Nanoparticles (ZnNPs, MnNPs, and SnNPs)

#### 2.3.1. FT-IR Spectroscopy

FT-IR spectrum of the CS capped by the ZnNPs as a represented sample revealed the different absorption bands as follows: 701.80 cm^−1^ ((CH_2_)n rocking), 1072.25 cm^−1^ (C-N+), 1375.49 cm^−1^ (CH_3_ symmetric bending), 1463.71 cm^−1^ (CH_2_ asymmetric bending), 1635.63 cm^−1^ (C=N), 2853.26 cm^−1^ (CH symmetric stretching) and 2924.44 cm^−1^ (CH asymmetric stretching), 3418.75 cm^−1^ (OH stretching) (see Figure S3).

#### 2.3.2. TEM

**Figure 2:**
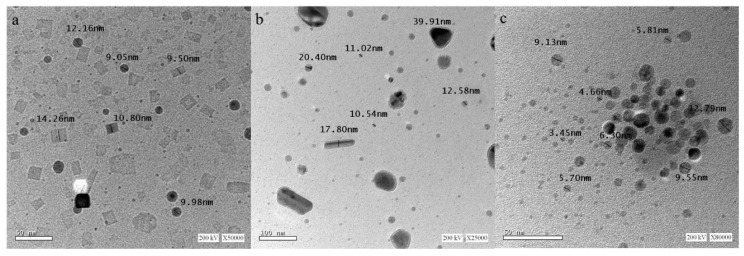
TEM images of the different nanoparticles capped by the synthesized cationic surfactant zinc, manganese and tin nanoparticles (**a**, **b** and **c**, respectively) in an aqueous phase.

The TEM analyses of the ZnNPs, MnNPs, and SnNPs capped by the CS are displayed Figure 2a–c, respectively. The TEM images demonstrated the self-assembling of the CS on the zinc, manganese, and tin nanoparticles. This form leads to more stabilization of these nanoparticles [23].

### 2.4. Surface Characteristics of the Synthesized CS

#### 2.4.1. The Surface Tension

**Figure 3:**
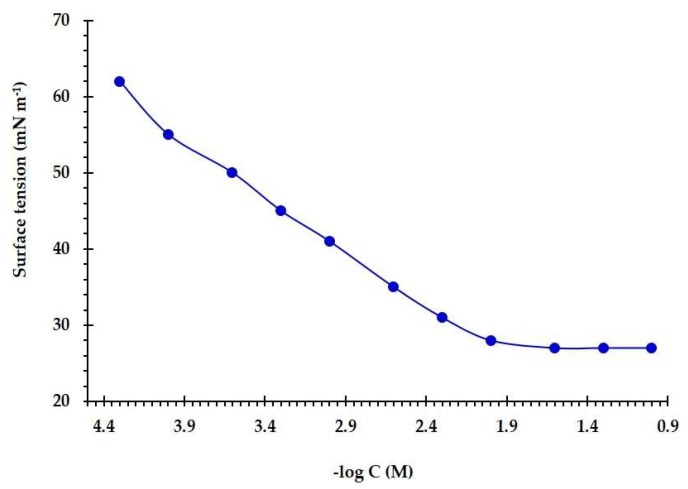
Variation of the surface tension values with application of ((*Z*)-2-((1-methyl-1-dodecyl pyrrolidin-1-ium-2-ylidene)amino)ethan-1-ol bromide at different concentrations in water at 25 °C.

The surface tension (γ) of the CS was measured for a range of concentrations above and below the critical micelle concentration (*C*_cmc_). Figure 3 displayed the surface tension (γ) versus the concentration of the (*Z*)-2-((1-methyl-1-dodecyl pyrrolidin-1-ium-2-ylidene)amino)ethan-1-ol bromide. It is clear that there is a linear decrease in surface tension values with increasing surfactant concentration up to the critical micelle concentration (*C*_cmc_).

The area per molecule, effectiveness, and surface concentration were obtained from the surface tension plot at the air–water interface. The effectiveness (*π*_cmc_) of a surfactant at *C*_cmc_ can be calculated as follows [24]:

(1) $$\pi_{\mathrm{cmc}}={\;\gamma }_{o}-{\;\gamma }_{\mathrm{cmc}}$$

where γ_o_ is the pure water surface tension and γ_cmc_ is the surface tension at *C*_cmc_.

Maximum surface excess of a surfactant (Γ_max_) at the interface is obtained from the Gibbs adsorption equation [25]:

(2) $$\Gamma_{\max}=\left(\frac{-1}{\mathrm{nRT}}\right)\left(\frac{d\gamma }{d\ln C}\right)$$

where C is concentration; R is gas constant; T is absolute temperature, and *n* is the number of species at an interface.

The minimal surface area per adsorbed molecule (A_min_) (nm^2^), is calculated as follows [26]:

(3) $$A_{\min}=\frac{10^{14}}{N_{A}\Gamma_{\max}}$$

where N_A_ is the Avogadro number and Γ_max_ (mol cm^−2^) is the maximal surface excess of adsorbed surfactant.

The values of C_cmc_, π_cmc_, Γ_max_ and A_min_ were estimated and reported in Table 1. These results revealed that the present CS has a strong surface activity directed to the air/water interface.

The C_cmc_ is representing the stability of the surfactant micellar. The ΔG^o^_m_ of the CS can be estimated from the next equation [27]:

(4) $$\Delta G_{m}^{o}=\left(2-\;\beta \right)\mathrm{RT}\ln C_{\mathrm{cmc}}$$

where R is the gas constant, T is the temperature and β is the degree of counter ion dissociation. The obtained ΔG^o^_m_ was calculated and reported in Table 1. The negativity of the obtained value (−22.68 kJ mol^−1^) indicated the tendency of the CS to be adsorbed at the interface.

#### 2.4.2. Conductivity

**Figure 4:**
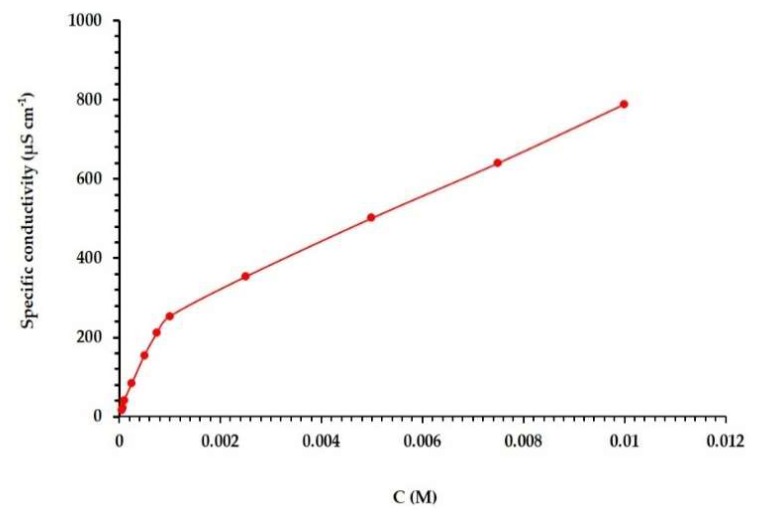
Plot of electrical conductivity against concentration of (*Z*)-2-((1-methyl-1-dodecyl pyrrolidin-1-ium-2-ylidene)amino)ethan-1-ol bromide in water at 25 °C.

Measuring the conductivity (K) of the CS is important to confirm the measured *C*_cmc_ from the surface tension plot. It can also be used to estimate the degree of counter ion dissociation (β) at 25 °C. It has been previously reported that the measured conductivity is linearly related to the concentration of surfactants (in pre-micellar and post-micellar areas). Moreover, it is well known that the specific conductivity is linearly correlated with the surfactant concentration in both the pre-micellar and post-micellar regions, and that the slope in the pre-micellar areas is remarkably higher than that in the post-micellar region (see Figure 4) [28]. The counter ion dissociation (β) is obtained from the ratio between the post-micellar and the premicellar areas (Table 1). The C_cmc_ value determined from the conductivity slope was in agreement with that obtained from the surface tension slope.

### 2.5. Antimicrobial Activities of the CS and the Different Nanoparticles Capped by the CS

The results (Table 2) displayed the broad-spectrum antimicrobial effect of the synthesized CS and the ZnNPs-C-CS, MnNPs-C-CS, and SnNPs-C-CS. All the tested compounds exhibited higher antimicrobial activities in comparison to the positive controls used. Additionally, all the nanoparticles capped by the CS displayed higher anti-antimicrobial activities than the synthesized CS alone. Furthermore, the SnNPs-C-CS displayed the highest antimicrobial activities in comparison to the MnNPs-C-CS and the ZnNPs-C-CS. This result may be attributed to the fact that the synthesized Sn nanoparticle showed the smallest crystallite size (see Figure 2c), which easily penetrates the microbial cells via the effect of electrostatic interactions (formed between the ions and the microbial cell) [29]. This effect may also be related to changes in the potentiality and reactivity of the synthesized nanoparticles in the electrochemical series. The results also displayed that all the nanoparticles capped by CS and the CS itself displayed higher distinct antibacterial activities for Gram-positive bacteria (32.9–71.8 mm) than Gram-negative bacteria (27.0–62.0 mm). This activity may be attributed to the distinct structural difference between Gram-positive and Gram-negative bacteria in their cell wall, as previously reported [7]. Briefly, Gram-negative bacteria are completely different in their cell wall structure in comparison to Gram-positive bacteria. These differences can be explained by penetration and retention of most of the antimicrobial agents. Gram-negative bacteria possess an envelope that is composed of three principal layers. The first layer is the outer membrane, which is unique and differentiates Gram-negative bacteria from Gram-positive bacteria. This layer serves as a permeability barrier and is composed mainly of phospholipids, lipopolysaccharide (LPS) and outer membrane proteins. The second layer is composed of the thin layer cell-wall peptidoglycan (~10 nm). The third layer is called the cytoplasmic or inner membrane, which is a phospholipid bilayer. The outer membrane of Gram-negative bacteria is the main reason for resistance to a wide range of antimicrobial agents in comparison to Gram-positive bacteria, which lack this outer membrane [30,31].

In addition, the synthesized CS and the nanoparticles capped by the CS exhibited higher activities against bacteria (27.0–71.8 mm) in comparison to their activities against the yeast strain (19.8–60.0 mm).

*Pseudomonas* strains (Gram-negative, rod-shaped, motile, and aerobic bacteria) have been repeatedly detected in a marine-induced corrosion medium that induces severe damage and huge economical impacts [32,33]. Accordingly, the present work aimed to evaluate the CS and the nanoparticles capped by the CS as potential biocides against the environmentally isolated *Pseudomonas* and, consequently, as corrosion inhibitors under high salinity (3.5% NaCl). Results for the antibacterial activity of the synthesized compounds against *Pseudomonas* sp. (R301) at a salinity of 3.5% NaCl showed inhibition zones of 30.1, 60.5, 63.8 and 65.5 mm for the CS, CS-C-ZnNPs, CS-C-MnNPs, and CS-C-SnNPs, respectively, compared to Benzalkonium chloride (50 ppm) with an inhibition zone of 32 mm (Table 2). In general, the highest antimicrobial activities were displayed in an order of SnNPs-C-CS > MnNPs-C-CS > ZnNPs-C-CS > CS.

The lowest MIC/MBC and MIC/MFC were achieved for the SnNPs-C-CS, with values of (0.078–0.156/0.078–0.234 mM and MIC/MFC of 0.156/0.156 mM) in comparison to the MnNPs-C-CS (with MIC/MBC of 0.156–0.117/0.156–0.312 mM and MIC/MFC of 0.312/0.312 mM), the ZnNPs-C-CS (with MIC/MBC of 0.156–0.312/0.156–0.312 mM and MIC/MFC of 0.312/0.625 mM) and the CS (with MIC/MBC of 0.312–0.625/0.312–1.25 mM and MIC/MFC of 1.25/1.25 mM) (see Table 3).

The results of MIC/MBC of the CS, ZnNPs-C-CS, MnNPs-C-CS, and SnNPs-C-CS against the isolated *Pseudomonas* sp. (R301) at a salinity of 3.5% ranged from 0.078–0.312 mM/0.156–0.312 mM (Table 3), with the highest values achieved for the SnNPs-C-CS. This means that the nanoparticles that were capped by the CS enhanced the antibacterial activity of the synthesized CS.

### 2.6. Anti-Biofilm Activities of the CS and Different Nanoparticles Capped by the CS

One of our aims was studying the anti-biofilm potentialities of the synthesized CS and the nanoparticles capped by the CS against developed biofilms of *B. subtilis* (ATCC 6633) and *E. coli* (ATCC 8739). The results in Table 4 represent the Minimum Biofilm Inhibitory Concentrations (MBICs) at a range of 0.156–1.25 mM against *B. subtilis*-developed biofilms and at a range of 0.156–1.25 mM against *E. coli*-developed biofilms. The highest efficiency was achieved for MnNPs-C-CS and SnNPs-C-CS. This result may be attributed to the effect of the synthesized CS in addition to the impact of the different nanoparticles, which easily penetrated the biofilms’ network and structure [20]. The suggested nanoparticle–biofilm interactions are related to the three main sequential actions: (i) transportation of NPs to the biofilm–fluid interface; (ii) attachment of the NPs to the outer region of the biofilms; and (iii) migration of the NPs within the biofilms’ matrices [34]. Additionally, the physicochemical features of the synthesized nanoparticles, such as their size, shape, surface charge, functional groups, and hydrophobicity, also reflect their potential against biofilm development [35].

### 2.7. Biocidal and Anti-Corrosion Susceptibility of the CS and the Different Nanoparticles Capped by the CS against SFB at a Salinity of 3.5% NaCl

**Figure 5:**
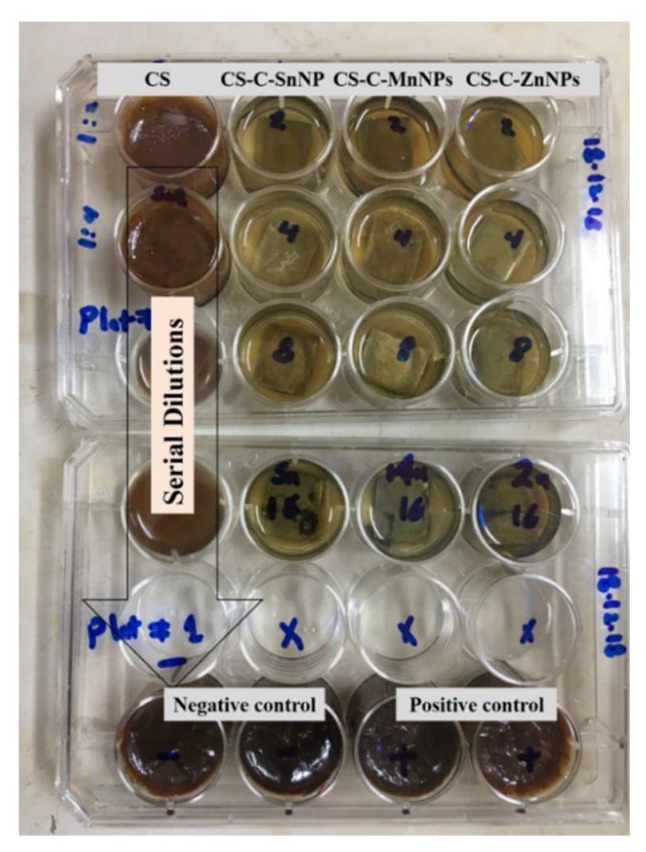
Photo documenting the anti-corrosion efficiency of CS, ZnNPs-C-CS, MnNPs-C-CS and SnNPs-C-CS against the isolated *Pseudomonas* sp. (R301), grown at a salinity of 3.5% NaCl in a 12-well microtiter plate, in comparison to the negative control (un-inoculated modified NRSS medium) and the positive control (the medium inoculated with *Pseudomonas* sp. (R301), without the synthesized compounds).

Furthermore, CS, ZnNPs-C-CS, MnNPs-C-CS, and SnNPs-C-CS were applied in a batch experiment to estimate their metal corrosion inhibition efficiencies on the carbon steel coupons under a cultivation salinity of 3.5% NaCl. The results (Table 5 and Figure 5) showed that the metal corrosion rate of the negative control (media with a salinity of 3.5% NaCl) was 1.94 g/m^2^ d. The negative control corrosivity can be attributed to the chloride anions’ chemisorption effect on the metallic materials. Such anions invaded the oxide film (via any pores or defects) that increasingly developed on the metallic materials and subsequently dispersed the oxide film. Moreover, the chloride anions favor the hydration of the metal ions, which further leads to pitting or crevice corrosion [36,37,38].

The corrosive *Pseudomonas* sp. (R301) covered the metal surface and protected it from the salinity corrosion effect with a corrosion inhibition efficiency (*IE*) of 27.4%, which is in agreement with the previously reported results [39]. This effect was explained by the ability of the *Pseudomonas* species to grow on the metal surface in a heterogeneous biofilm layer, which leads to the development of differential aeration cells between anodic and cathodic areas on the metallic surface. The anodic area is underneath the biofilm layer at a low oxygen concentration, whereas the cathodic area is allocated on the metallic surface at a high oxygen concentration. This oxygen concentration difference at the anodic and the cathodic areas induces activation of the electrochemical cells, which leads to severe pitting or crevice corrosion [40]. Moreover, *Pseudomonas* species were also involved in solubilization of the protective ferric iron layer (reduce ferric ions to ferrous), which exposes the metal to further oxidation, as reported previously [41].

Application of the CS, ZnNPs-C-CS, MnNPs-C-CS, and SnNPs-C-CS to the metal surface at a concentration of 1.25 mM increased inhibition of metal corrosion with *IE* of 85.7, 88.6, 94.0 and 96.9%, respectively. The corrosion inhibition mechanism of the CS is mainly attributed to its chemical structure and adsorption properties (protective films at the interface of the metal/liquid) [42]. The corrosion inhibition efficiency of the CS increased with capping with different nanoparticles. The corrosion inhibition effects can be attributed to the adsorption of the CS on the metallic surface by two mechanisms: physical and chemical adsorption. The physical adsorption of the CS happened when an electrostatic attraction between the group carrying charges and the charges of the metallic surface was formed. On the other hand, the chemical adsorption occurred when there was charge sharing between the surfactant molecule and the metallic surface [42,43]. The metal corrosion inhibition efficiencies of the CS were increased by capping different NPs, and this effect may be attributed to their properties [44,45].

Generally, the basic antimicrobial activity in this study was mainly attributed to the effect of the CS, which was increased by the effect of the nanoparticles that capped the CS. The mechanism of the antibacterial activity of the synthesized CS was accredited to the proposed electrostatic interaction, which formed between the positively charged ammonium group (R_4_N^+^) of the synthesized CS and the negatively charged bacterial cell membrane lipoprotein which emerged with cell disruption [46,47,48]. Additionally, the hydrophobic part of the synthesized CS can supposedly pass through the cell membrane, producing damage to its selective permeability and, in consequence, the death of the cell [49]. Another suggested mechanism for the antibacterial activity of the synthesized CS is an influx of molecules of surfactant into the cell that interact with particular organelles like mitochondria and vacuoles [50]. However, the antifungal activity mechanism of the synthesized CS was supposedly attributed to its power to incorporate the fungal plasma membrane, leading its dysfunction [51]. It was also reported that the synthesized cationic surfactants attached to the cell surface of the fungal cells, changing the charge of the membrane to a positive charge [52]. Accordingly, this synthesized CS can easily penetrate the cell and interact with the membrane of the mitochondria, which leads to severe oxidative stress [53]. On the other hand, the antimicrobial activity of the CS increased remarkably after capping the CS by nanoparticles, which can be explained by two proposed effects: (a) the effect of the generated reactive oxygen species (ROS) “Super Oxide Radical (O_2_/h^−^), Hydroxyl Radical (h^+^/OH) and Hydrogen Peroxide (H_2_O_2_)” and (b) the effect of the deposited nanoparticles on the cell wall of the microbial cell [14]. The potential effect of the generated H_2_O_2_ on the microbial cell leads to death, as previously reported [54,55]. Furthermore, the highest antimicrobial activities were displayed in an order of SnNPs-C-CS > MnNPs-C-CS > ZnNPs-C-CS > CS. This ordering of the synthesized nanoparticles may be attributed to their shapes and sizes, as displayed in TEM photos (see Figure 2). This means the highest antimicrobial activity, of the SnNPs-C-CS, was related to its spherical shape, with the smallest size in comparison to MnNPs-C-CS (an almost spherical nanoparticle) and ZnNPs-C-CS (an almost cubic nanoparticle). Kim and his colleagues [56] have reported that the smallest spherical nanoparticles showed better antimicrobial activity in comparison to the cubic nanoparticles. It has been reported that the nanoparticles’ size is an important factor to kill bacterial systems, as it allows easily penetration and accumulation into the bacteria which further leads to damage and death [57]. Furthermore, the smallest nanoparticle size has the highest surface area and the highest surface reactivity. In addition, when such nanoparticles are in the form of metal oxide nanoparticles with small size, this promotes easily permeability into bacterial cells [57]. Concerning the nanoparticles’ shapes, it depends on the synthesis method. There are a lot of studies that have reported many spherical nanoparticles with high antimicrobial activity [58,59].

### 2.8. Comparison of Inhibition Efficiencies between the Synthesized Cationic Surfactant (CS) and Other Cationic Surfactants

The inhibition efficiency of the synthesized cationic surfactant (CS) was compared to previously reported cationic surfactants [60,61,62,63] (see Table 6).

## 3. Mate rials and Methods

### 3.1. Synthesis of a Cationic Surfactant (CS)

**Figure 6:**
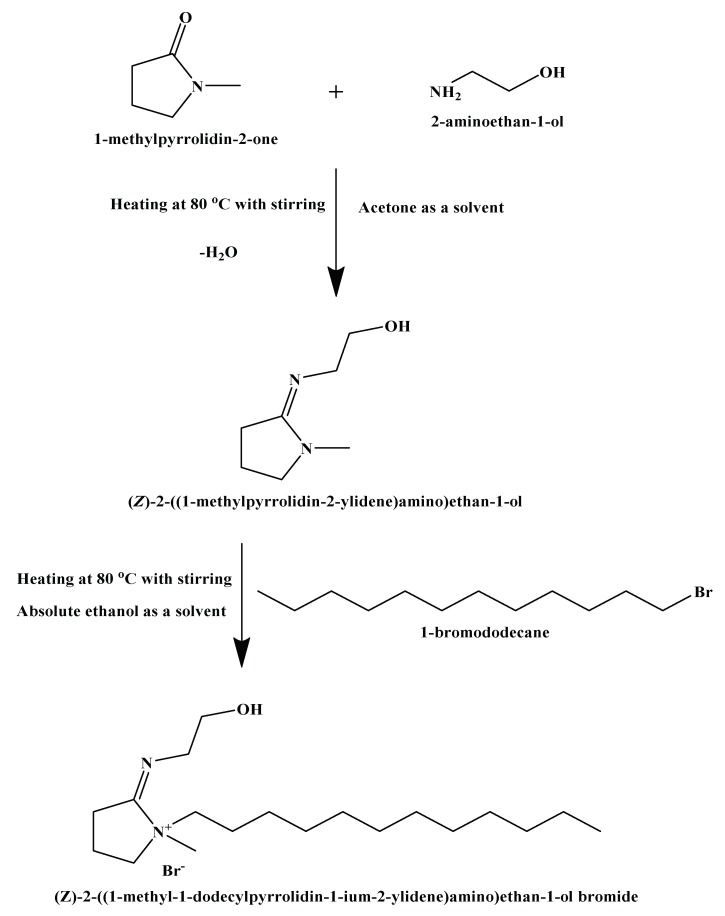
Preparation scheme of the synthesized cationic surfactant (CS).

In this study, a cationic surfactant was prepared according to the scheme in Figure 6. This scheme was accomplished in two main reactions as follows: (i) reaction of 2-aminoethan-1-ol (2 mol, 12.216 g) with 1-methylpyrrolidin-2-one (2 mol, 19.826 g) through condensation reaction with acetone (solvent) at 80 °C, with stirring for 6 h [64]. The reaction was then cooled down till all solvent evaporated completely. The obtained light viscous liquid was purified by diethyl ether and then recrystallized using ethanol [28]. The product was (*Z*)-2-((1-methylpyrrolidin- 2-ylidene)amino)ethan-1-ol. (ii) Reaction of (*Z*)-2-((1-methylpyrrolidin-2-ylidene) amino)ethan-1-ol (1 mol, 14.22 g) with 1-bromododecane (1 mol, 24.923 g) through quaternization reaction in absolute ethanol (solvent) at 80 °C, with stirring for 24 h [65]. The product was cooled down till all solvent evaporated completely, and the obtained pale brown viscous liquid was purified by diethyl ether and then recrystallized by absolute ethanol. The final product was (*Z*)-2-((1-methyl-1-dodecylpyrrolidin-1-ium-2-ylidene)amino) ethan-1-ol bromide.

The structure of the resulted compound was elucidated by Fourier-Transform Infrared (FT-IR) and Nuclear Magnetic Resonance (NMR) spectroscopy analyses.

### 3.2. Preparation and Capping of Zinc, Manganese and Tin Nanoparticles (ZnNPs, MnNPs, and SnNPs) on the Synthesized CS

Zinc, manganese, and tin nanoparticles were synthesized via reduction reactions of ZnCl_2_, MnCl_2_, and SnCl_2_ by NaBH_4_ in a solution of the CS through the following steps. A solution of metals chloride (0.01 M) was prepared and added to a solution of ascorbic acid (0.1 M) as an antioxidant of colloidal metals with stirring for 30 min.

(i) A dried amount of the synthesized cationic surfactant (as a capping agent) was supplemented to the previous mixture.
(ii) About 0.1 M of NaOH solution was added dropwise to achieve pH 12, and the solution was stirred for 1 h. at room temp.
(iii) A solution of NaBH4 (0.1 M) was supplemented (as a reducing agent) to the reaction under continuous vigorous stirring for 10 min.
(iv) The metal nanoparticles capped by the CS were collected by centrifugation and washed with deionized water. Acetone was used to remove the excess cationic surfactants.
(v) The resultant precipitates were dried under vacuum for 3–4 h [66].

Characterization of the ZnNPs, MnNPs, and SnNPs capped by the CS was confirmed by Fourier-Transform Infrared (FTIR) spectroscopy and Transmission Electron Microscopy (TEM).

### 3.3. Surface Tension Estimation of the Synthesized CS and Its Surface Characteristics

Surface tension was obtained by Krüss-K6 tensiometer (Krüss Company, Hamburg, Germany) via applying the ring method at 25 °C. The surface tension of pure water was used for the calibration. The effectiveness (π_cmc_) at C_cmc_, the maximum surface excess (Γ_max_), the minimal surface area per adsorbed molecule (A_min_), the degree of counter ion dissociation (β) and the free energy of micellization (ΔG^o^_m_) of the synthesized surfactant were calculated from the surface tension values.

### 3.4. Conductivity Estimation of the Synthesized CS

Conductivity was explored with a digital conductivity meter (Type 522; Crison Instrument, S.A., Barcelona, Spain) in a double jacket glass cell at 25 °C in order to confirm the surface tension results.

### 3.5. Antimicrobial Activities of the CS and the Different Nanoparticles Capped by the CS aginst Standard Microbial Strains and Slime-Forming Bacteria (SFB) at a Salinity of 3.5% NaCl

#### 3.5.1. Microbial Strains

The standard microbial strains in this study were *Staphylococcus aureus* (DSM 3463) and *Bacillus subtilis* (ATCC 6633) as Gram-positive bacterial strains; *Escherichia coli* (ATCC 8739) and *Pseudomonas aeruginosa* (ATCC 9027) as Gram-negative bacterial strains and *Candida albicans* (IMRU3669).

In this study, *Pseudomonas* sp., a slime-forming bacterium (SFB), was isolated from the formation water of General Petroleum Company, Ras Gharib, Egypt on a Cetrimide agar (Sigma-Aldrich) at a salinity of 3.5% NaCl. The bacteria were purified, identified using 16S rRNA (data not shown), and have been deposited in the GenBank (R301).

#### 3.5.2. Cultivation Media

Mueller Hinton Agar (MHA) and Mueller Hinton Broth (MHB) (Difco, Franklin Lakes, NJ, USA) were used for the cultivation of the standard bacterial strains. Sabouraud Dextrose Agar (SDA) and Sabouraud Dextrose Broth (SDB) (Difco, Sparks, MD, USA) were used for the cultivation of the standard yeast strain. Furthermore, Mueller Hinton Agar (MHA) and Mueller Hinton Broth (MHB) (Difco, Franklin Lakes, NJ, USA) supplemented with 3.5% NaCl were used for the cultivation of isolated *Pseudomonas* sp. (R301).

#### 3.5.3. Agar Well Dfffusion Method

The antimicrobial activities of the CS and the different nanoparticles capped by the CS (ZnNPs-C-CS, MnNPs-C-CS, and SnNPs-C-CS) were determined by the agar well diffusion method as previously described [67], at a concentration of 10 mM on strain’s agar-specific media. All the microbial strains were streaked on their relative agar plates. The CS and the different nanoparticles capped by the CS (ZnNPs-C-CS, MnNPs-C-CS, and SnNPs-C-CS) were added to the wells. Then, the plates were incubated overnight at 37 °C in the case of bacterial strains and for 48 h at 30 °C in the case of the yeast strain. The diameters of the clearing zones were measured to indicate the antimicrobial susceptibility. This test was done in triplicate, and the average value was obtained. This test was run in comparison to a negative (sterile water) control and positive controls (standard antibiotics, amoxicillin (0.273 mM), tetracycline (0.225 mM), fluconazole (0.326 mM) for standard Gram-positive bacteria, Gram-negative bacteria, and yeast strains, respectively. Benzalkonium chloride (0.138 mM) was used as a poistive control for *Pseudomonas* sp. (R301).

#### 3.5.4. Minimum Inhibitory Concentration and Minimum Bactericidal or Fungicidal Concentrations

The minimum inhibitory concentrations (MIC)s were determined by the micro-serial dilution method in 96-well microtiter plates (Nunc GmbH & Co., Wiesbaden, Germany) [68,69,70]. The bacterial strains were refreshed on 10 mL of MHB and MHB with 3.5% NaCl for the standard bacterial strains and the isolated *Pseudomonas* sp. (R301), respectively, and incubated at 37 °C for an overnight incubation period with agitation of 200 rpm. These overnight cultures were re-cultivated again with 10 mL of the same media by adjusting the optical density (OD) to 0.2 at 550 mm and incubated for 3–4 h at 37 °C to achieve the OD550 of 1–2. Afterwards, the cultivated media were diluted to obtain OD550 of 0.2. The bacterial inocula were diluted again with the sterile media by 100-fold dilution (DF) and 1000-fold dilution for Gram-positive and Gram-negative bacteria, respectively, to achieve bacterial counts of 1–2 × 10^8^ CFU/mL for Gram-positive bacteria and 1–2 × 10^9^ CFU/mL for Gram-negative bacteria, according to the Clinical Laboratory Standards Institute (CLSI) [71]. The yeast stain (*Candida albicans*) inocula was freshly prepared on 10 mL of SDB and incubated at 30 °C for 24 h under continuous agitation at 200 rpm. Afterwards, the cultivated cells were diluted with sterile SDB to achieve OD to 550 to 0.2 according to the Clinical Laboratory Standards Institute (CLSI), which corresponded to the candida count of 5 × 10^6^ CFU/mL [72]. A quantity of 100 µL of the CS and the ZnNPs-C-CS, MnNPs-C-CS, SnNPs-C-CS at a concentration of 10 mM was serially diluted with MHB and SDB for the bacterial and the yeast standard strains, respectively, and MHB with 3.5% NaCl for the isolated *Pseudomonas* sp. (R301) onto the micro-titer plates. The microtiter plates were also inoculated with a positive control (only the microbial strains) and a negative control (only sterile media). The result was recorded after 20 h at 37 °C for the bacterial strains and 72 h at 30 °C for the yeast strain. This experiment was visually observed and further confirmed using 30 µl of 0.01% resazurin (HiMedia) solution [73] after incubating the plates for 3 h. The result was considered as positive by observing the changing of the well’s color to pink. MBC/MFC values of the CS and the ZnNPs-C-CS, MnNPs-C-CS and SnNPs-C-CS were obtained by taking a sample (10 µL) from the wells (before resazurin indicator) displaying no distinguishable microbial growth onto their specific agar media (MHA, SDA plates) [74]. The result was observed after 20 h at 37 °C in the case of bacterial strains and after 48 h at 30 °C in the case of *C. albicans*.

### 3.6. Anti-Biofilm Activities of the CS and the Different Nanoparticles Capped by the CS

The anti-bacterial biofilm (*B. subtilis* and *E. coli*) activity of the CS and the ZnNPs-C-CS, MnNPs-C-CS and SnNPs-C-CS was estimated with a semi-quantitative adherence assay in 96-well microtiter plates as previously described [68,69,70]. Quantities of 100 µls of the CS and the ZnNPs-C-CS, MnNPs-C-CS and SnNPs-C-CS (concentration of 10 mM) were serially diluted on MHB medium supplemented with 1% glucose onto the microtiter plate (Nunc GmbH & Co., Wiesbaden, Germany) with a flat surface. The plate’s inocula were 100 µL, which contained 1–2 × 10^8^ CFU/mL and 1–2 × 10^9^ CFU/mL for *B. subtilis* and *E. coli*, respectively, as previously recommended [71]. A positive control (inoculated well without the synthesized products) and negative control (only sterile medium) were also prepared in the same plate. After incubation (20 h at 37 °C), the planktonic suspension with non- adherent cells and nutrient solution should be removed. Afterwards, the plate was cleaned with 200 μL of 1× phosphate buffer saline (PBS) at a pH of 7.4, dried, fixed with 97% ethanol, then stained with 1.0% crystal violet (Merck, Darmstadt, Germany) for 5 min. The stained plates were washed in running tap water and dried for 2 h [70]. Then, the developed positive result appeared as purple color rings on the well’s bottom and side. The result can be quantified by adding 200 μL glacial acetic acid (Merck) at a concentration of 33% (*v*/*v*) to the dried plate. Afterwards, the optical density of the plate was measured at 570 nm by an ELISA reader.

### 3.7. Biocidal and Anti-Corrosion Susceptibility of the CS and the Different Nanoparticles Capped by the CS against SFB at a Salinity of 3.5% NaCl

This experiment was carried out using batch cultivation of the nutrient-rich simulated seawter (NRSS) at the medium salinity [75]. The inocula for the corrosion experiment were enriched for 20 h at 37 °C using a Cetrimide broth medium (Sigma-Aldrich) at a salinity of 3.5% NaCl. The test was initiated on mild steel coupons with a chemical composition represented in Table 7 (AISI 1018 mild carbon steel strip (1.5 × 1.5 × 0.32cm), COSASCO’s, Rohrback Cosasco Systems, Inc), in a 12-well plate (Nunc GmbH & Co., Wiesbaden, Germany). The mild steel coupons were primarily cleaned up mechanically using various emery papers and then washed vigorously with deionized water and acetone. The cleaned coupons were dried in a desiccator, under aspectic conditions, for further application. The weights of the coupons were achieved and aseptically added to the plates. The experiment was carried out at various concentrations of CS, ZnNPs-C-CS, MnNPs-C-CS and SnNPs-C-CS by a serial dilution method (1.25, 0.625, 0.312, 0.156 mM) in parallel to s negative control (un-inoculated modified NRSS medium) and a positive control (the medium inoculated with *Pseudomonas* sp. (R301), without the synthesized compounds). Afterwards, the plates were inoculated with the bacterial inocula with a count of 49 ×10^6^ CFU/mL (obtained by a serial dilution method). The plates were then incubated at 37 °C for 21 days. This test was in duplicate. At the end of the expermint, the coupons were taken from the plate and cleaned by Clarke solution (1 L 36% HCl, 20 g Sb_2_O_3_ and 50 g SnCl_2_) for 10−15 sec, deionized water, and ethanol, and then dried in the desiccator. The coupons’ weight loss was obtained by calculating the weight of the mild steel coupons before and after the experiment. The weight loss results were used in the calculation of the metal corrosion rate, *K* (g/m^2^ d) and the corrosion inhibition efficiency, IE (%) [76,77] as follows:

(5) $$k=\frac{\Delta W}{At}$$

(6) $$IE=\frac{\left(k_{1}-k_{2}\right)}{k_{1}}\times 100$$

where *ΔW* is the difference in weight of the mild steel coupons before and after the experiment (weight loss), *A* is the surface area in m^2^, *t* is the time in days, *k*_1_ is the corrosion rate for the negative control (un-inoculated modified NRSS medium), and *k*_2_ is the corrosion rate of the positive control (the medium inoculated with *Pseudomonas* sp. (R301), without the synthesized compounds), in the presence of the CS and the different nanoparticles capped by the CS.

## 4. Conclusions

(*Z*)-2-((1-methyl-1-dodecylpyrrolidin-1-ium-2-ylidene)amino)ethan-1-ol bromide as a pyrrolidinium-derivative cationic surfactant (CS) was successfully synthesized and characterized using Fourier-Transform Infrared (FTIR) and Nuclear Magnetic Resonance (NMR) spectroscopy, and its surface properties were calculated. The efficiency and applicability of the CS were improved through synthesis of different nanoparticles: zinc, manganese, and tin (ZnNPs, MnNPs, and SnNPs) that capped by the CS (ZnNPs-C-CS, MnNPs-C-CS, and SnNPs-C-CS). The ZnNPs-C-CS, MnNPs-C-CS, and SnNPs-C-CS displayed higher anti-microbial activities (against Gram-positive and Gram-negative bacteria, and yeast standard strains), with lower MIC and MFC/MBC, and higher anti-biofilm activity (against aerobic developed bacterial biofilms) with lower MBIC than the CS, and the activities were arranged in the order: SnNPs-C-CS > MnNPs-C-CS > ZnNPs-C-CS > CS. Although there is scarce information on cationic surfactants’ biocidal and corrosion inhbibition effects on Slime-Forming Bacteria (SFB) isolated from oil field formation water, the CS and ZnNPs-C-CS, MnNPs-C-CS and SnNPs-C-CS exhibited biocidal activities against the SFB and corrosion inhibitors at their cultivated salinity. Interestingly, the results revealed a strong effect of the different NPs capped by CS at lower concentrations as metal corrosion inhibitors, with efficiencies of 88.6, 94.0 and 96.9% in comparison to the CS efficiency of 85.7%.

## Figures and Tables

**Table 1:** Critical Micelle Concentration (C_cmc_), effectiveness (π_cmc_), maximum surface excess (Γ_max_), minimum area (A_min_), the degree of counter ion dissociation (β) and free energy of micellization (ΔG^o^_m_) of the synthesized CS from surface tension measurements at 25 °C.

C_cmc_ mol dm^−3^	γ_cmc_ mN m^−1^	π_cmc_ mN m^−1^	Γ_max_ × 10^10^ mol cm^−2^	A_min_ nm^2^	β	ΔG^o^_m_ (kJ mol^−1^)
0.0073	27.3	44.7	5.11	0.32	0.21	−22.68

**Table 2:** The antimicrobial activities of the synthesized CS and the different nanoparticles capped by the CS (ZnNPs-C-CS, MnNPs-C-CS, and SnNPs-C-CS). The results are the mean of diameters of the inhibition zones (mm).

Compounds	*Staphylococcus aureus* (DSM 3463)	*Bacillus subtilis* (ATCC6633)	*Escherichia coli* (ATCC 8739)	*Pseudomonas aeruginosa* (ATCC 9027)	*Candida albicans* (IMRU3669)	*Pseudomonas* sp. (R301)
	Mean Inhibition zone (mm)					
CS	35.3 ± 0.7	32.6 ± 0.5	29.6 ± 0.2	27.0 ± 0.5	19.8 ± 0.2	30.1 ± 0.2
ZnNPs-C-CS	55.3 ± 0.2	49.8 ± 0.7	47.2 ± 0.4	45.1 ± 0.2	48.0 ± 0.0	60.5 ± 0.8
MnNPs-C-CS	63.8 ± 1.0	61.3 ±1.1	54.8 ± 0.2	58.6 ± 1.5	50.8 ± 1.0	63.8 ± 0.7
SnNPs-C-CS	71.8 ± 0.7	65.1 ± 0.7	62.0 ± 0.0	60.6 ± 0.5	60.0 ± 1.0	65.5 ± 0.5
* AMC	20.0 ± 0.0	17.0 ± 0.0	ND	ND	ND	ND
* TE	** ND	ND	22.0 ± 0.0	23.5 ± 0.7	ND	ND
* Flu	ND	ND	ND	ND	17.0 ± 0.2	ND
* BAC	ND	ND	ND	ND	ND	31.0 ± 1.0

* AMC, Amoxicillin (0.273 mM), TE, Tetracycline (0.225 mM), Flu, Fluconazole (0.326 mM), BAC, Benzalkonium chloride (0.138 mM). ** ND, not detected.

**Table 3:** The Minimum Inhibitory Concentrations (MICs), the Minimum Bactericidal Concentrations (MBCs) and the Minimum Fungicidal Concentrations (MFCs) of the synthesized CS and the different nanoparticles capped by the CS (ZnNPs-C-CS, MnNPs-C-CS and SnNPs-C-CS). The results are represented as the mean of the concentrations (mM).

Compounds	*Staphylococcus aureus* (DSM 3463)		*Bacillus subtilis* (ATCC 6633)		*Escherichia coli* (ATCC 8739)		*Pseudomonas aeruginosa* (ATCC 9027)		*Candida albicans* (IMRU3669)		*Pseudomonas* sp. (R301)	
	MIC	MBC	MIC	MBC	MIC	MBC	MIC	MBC	MIC	MFC	MIC	MBC
CS	0.312	0.312	0.625	1.25	0.625	1.25	0.312	0.312	1.25	1.25	0.312	0.312
ZnNPs-C-CS	0.156	0.312	0.156	0.312	0.312	0.312	0.156	0.156	0.312	0.625	0.117	0.156
MnNPs-C-CS	0.117	0.312	0.156	0.234	0.156	0.156	0.156	0.234	0.312	0.312	0.078	0.156
SnNPs-C-CS	0.078	0.078	0.0780	0.117	0.156	0.234	0.117	0.156	0.156	0.156	0.078	0.156

**Table 4:** The Minimum Biofilm Inhibitory Concentrations (MBICs) of the synthesized CS and the nanoparticles capped by the CS (ZnNPs-C-CS, MnNPs-C-CS and SnNPs-C-CS) against two bacterial-developed biofilms. The results are represented as the mean of the concentrations (mM).

Samples	*Bacillus subtilis* (ATCC 6633)	*Escherichia coli* (ATCC 8739)
	MBIC	MBIC
CS	1.25	1.25
ZnNPs-C-CS	0.312	0.468
MnNPs-C-CS	0.156	0.156
SnNPs-C-CS	0.156	0.156

**Table 5:** The results of the corrosion rate, *K* (g/m^2^ d) and metal corrosion inhibition efficiency, *IE* (%) of the synthesized CS and the different nanoparticles capped by the CS (ZnNPs-C-CS, MnNPs-C-CS, and SnNPs-C-CS) against the isolated *Pseudomonas* sp. (R301) at 3.5% NaCl.

Samples	Concentration (mM)	Mean Corrosion Rate, *K* (g/m^2^ d)	Metal Corrosion Efficiency, *IE* (%)
Negative control	-	1.940 ± 0.020	0
Positive control	-	1.407 ± 0.070	27.4
CS	1.25	0.275 ± 0.010	85.7
	0.625	0.627 ± 0.002	67.6
	0.312	0.927 ± 0.010	52.2
	0.156	1.250 ± 0.090	35.5
ZnNPs-C-CS	1.25	0.221 ± 0.008	88.6
	0.625	0.431 ± 0.004	77.7
	0.312	0.602 ± 0.010	68.9
	0.156	0.819 ± 0.020	57.7
MnNPs-C-CS	1.25	0.115 ± 0.010	94.0
	0.625	0.368 ± 0.020	81.0
	0.312	0.542 ± 0.030	72.0
	0.156	0.767 ± 0.060	60.4
SnNPs-C-CS	1.25	0.059 ± 0.005	96.9
	0.625	0.158 ± 0.007	91.8
	0.312	0.549 ± 0.002	71.7
	0.156	0.647 ± 0.020	66.6

**Table 6:** Comparison of the inhibition efficiencies (*IE*) between the the present work’s synthesized surfactant and other previously published surfactants.

Cationic Surfactants	Concentration	Medium	*IE* (%)	Reference
*Z*)-2-((1-methyl-1-dodecylpyrrolidin-1-ium-2-ylidene)amino) ethan-1-ol bromide.	1.25 mM	Slime-forming bacteria in 3.5% (NaCl)salinity	85.7	[Present work]
Zinc nanoparticles capped by the CS; (*Z*)-2-((1-methyl-1- dodecylpyrrolidin-1-ium-2-ylidene)amino)ethan-1-ol bromide.	1.25 mM	Slime-forming bacteria in 3.5% (NaCl)salinity	88.6	
Manganese nanoparticles capped by the CS; (*Z*)-2-((1-methyl-1- dodecylpyrrolidin-1-ium-2-ylidene)amino)ethan-1-ol bromide.	1.25 mM	Slime-forming bacteria in 3.5% (NaCl)salinity	94.0	
Tin nanoparticles capped by the CS; (*Z*)-2-((1-methyl-1- dodecylpyrrolidin-1-ium-2-ylidene)amino)ethan-1-ol bromide.	1.25 mM	Slime-forming bacteria at 3.5% (NaCl) salinity	96.9	
2,2′-(1-aminopropane-1,3-diyl)bis(1-(2-aminoethyl)-1dodecyl-4,5-dihydro-1Himidazol-1ium)dichloride(I) and 2,2′-(1-aminoethane- 1,2 diyl)bis(1-(2aminoethyl)-1-dodecyl-4,5-dihydro-1H-imidazol-1ium)dichlor-ide(II)		Oilfield produced water under sweet conditions	83.1–88.1	[60]
1, 2-Ethanediylbis(alkyldimethylammonium) bromide R = 12, 14 and 16	6.0 mM	Seawater	94, 95.5 and 98.9%	[61]
Bis(2-hydroxy-3-(3-(dodecanoyloxy)propyl dimethylammonio) propyl) alkylamine dichloride R = 4, 6 and 8)	300 ppm	Formation water	76.9, 79.5 and 81.3%	[62]
tetradecyl dimethyl benzyl ammonium chloride (TDBAC) and tributyl tetradecyl phosphonium chloride (TTPC)	600 ppm	Oil-field water	93.43–64.83	[63]

**Table 7:** Chemical composition of a mild steel coupon AISI 1018 mild/low carbon steel strip COSASCO’s, Rohrback Cosasco Systems, Inc.

Element	Content
Carbon, C	0.14–0.20%
Iron, Fe	98.81–99.26% (as remainder)
Manganese, Mn	0.60–0.90%
Phosphorous, P	≤0.040%
Sulfur, S	≤0.050%

## Supplementary Materials

The following are available online, Figure S1. FT-IR spectrum of (*Z*)-2-((1-methyl-1-dodecylpyrrolidin-1-ium-2-ylidene)amino)ethan-1-ol bromide, Figure S2. 1H NMR spectrum of (*Z*)-2-((1-methyl-1-dodecylpyrrolidin-1-ium-2-ylidene)amino) ethan-1-ol bromide, Figure S3. FT-IR spectrum of the Zinc nanoparticles capped by the CS; (*Z*)-2-((1-methyl-1-dodecylpyrrolidin-1-ium-2-ylidene)amino)ethan-1-ol bromide.
