# Supplementary material for: Enhancement of A Cationic Surfactant by Capping Nanoparticles: Synthesis, Characterization and Multiple Applications
Source: Molecules. 2020 Apr 25;25(9):2007. doi: 10.3390/molecules25092007 (PMC7249094; doi:10.3390/molecules25092007)
Supplement: Supplementary file 1 [file molecules-25-02007-s001.pdf]

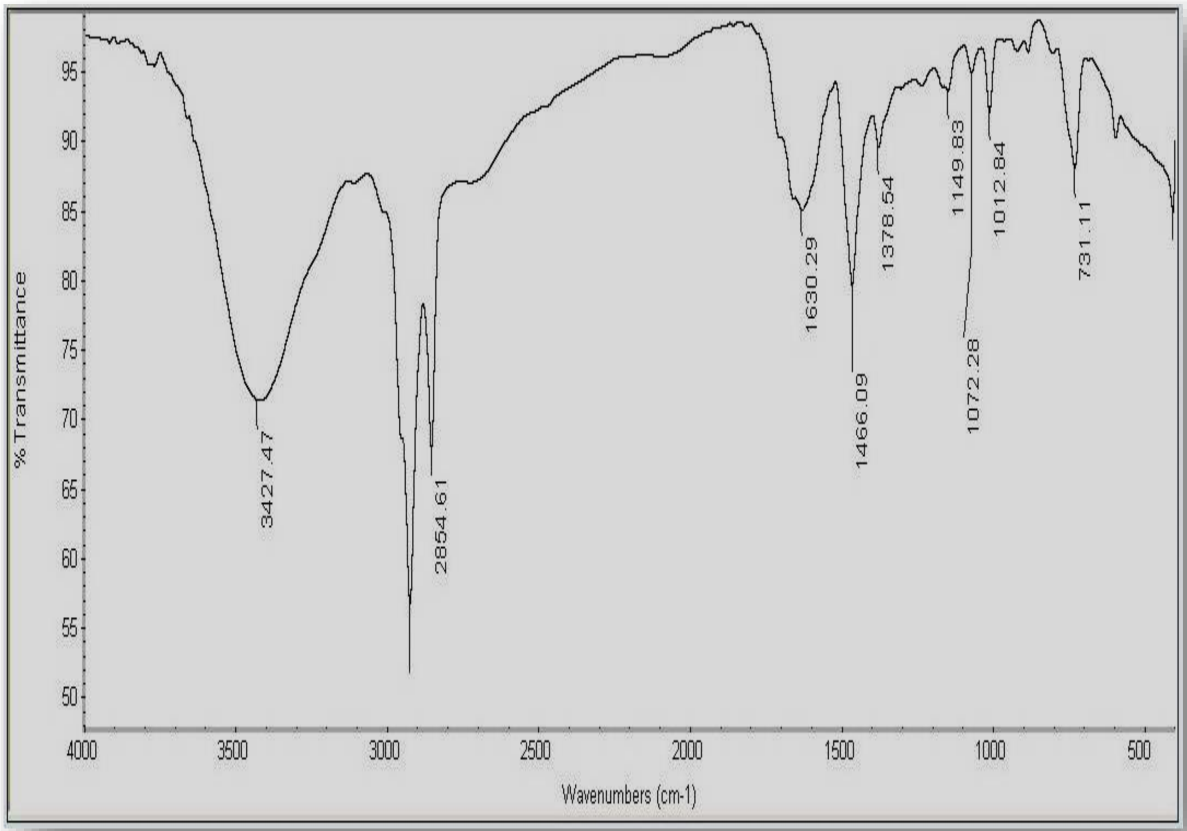

Figure S1. FT-IR spectrum of (Z)-2-((1-methyl-1-dodecylpyrrolidin-1-ium-2-ylidene)amino)ethan-1-ol bromide.

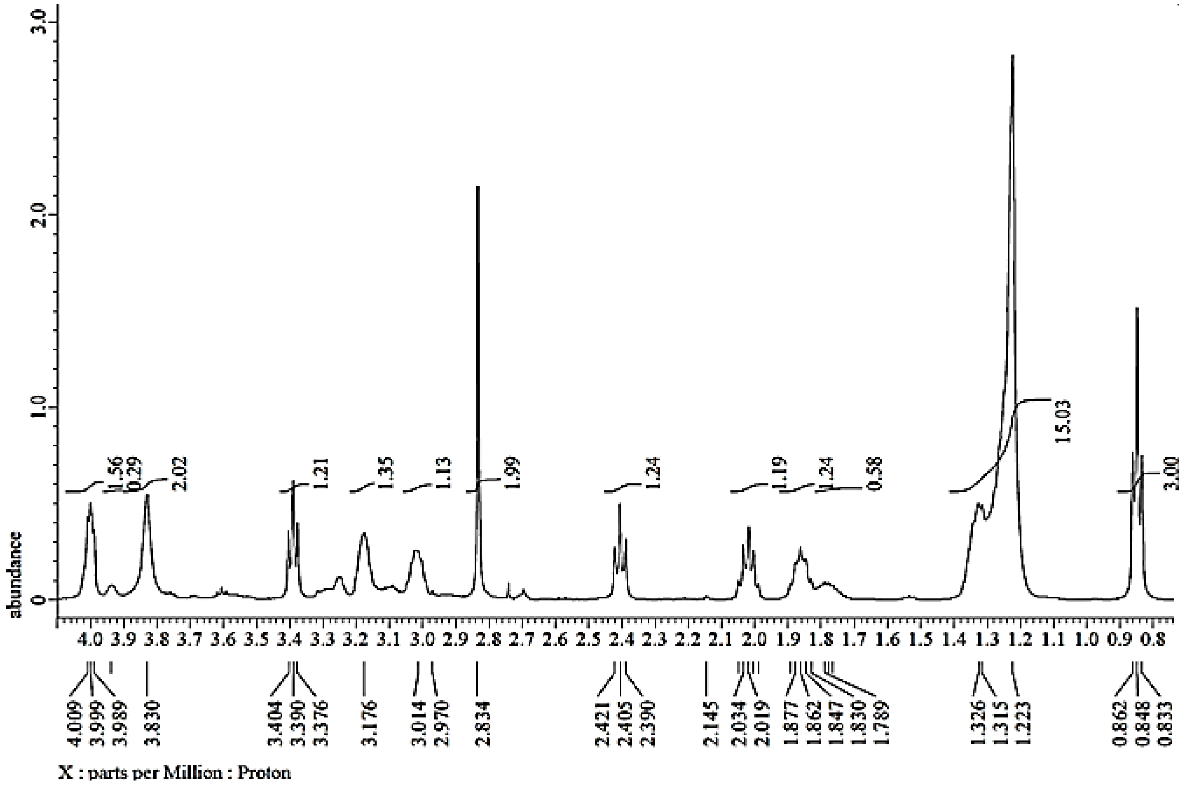

Figure S2. ^{1}H NMR spectrum of (Z)-2-((1-methyl-1-dodecylpyrrolidin-1-ium-2-ylidene)amino) ethan-1-ol bromide.

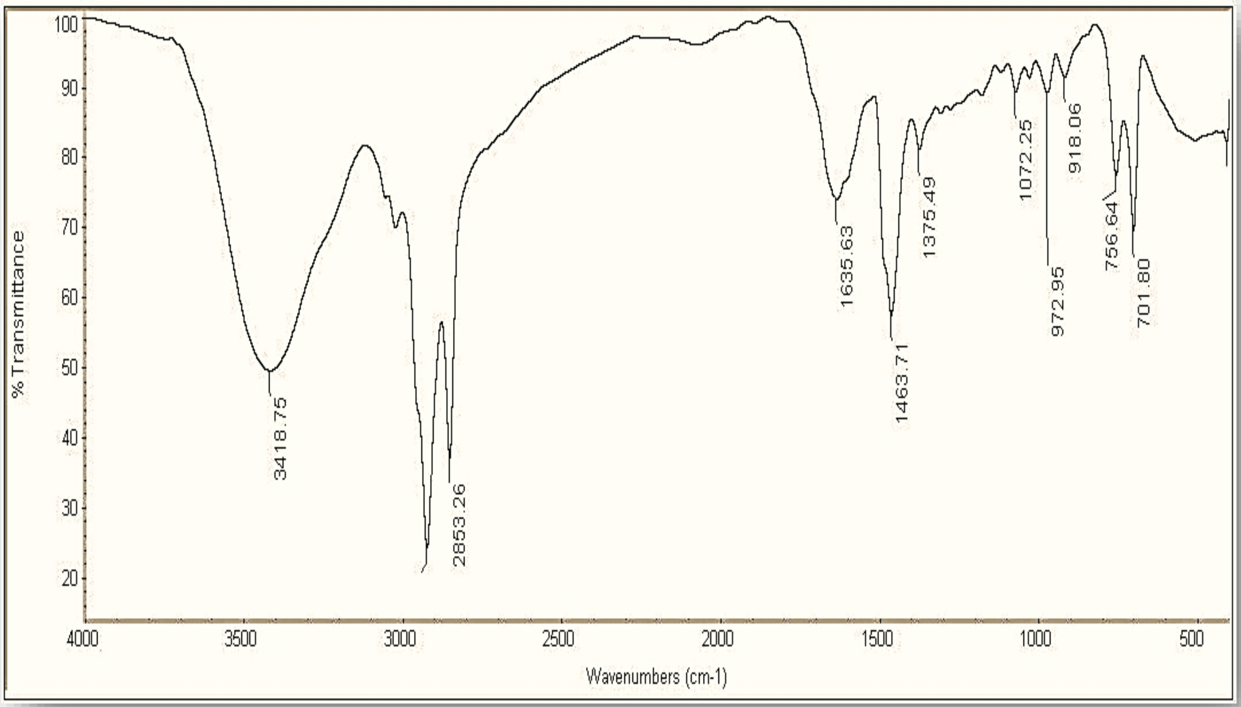

Figure S3. FT-IR spectrum of the Zinc nanoparticles capped by the CS; (Z)-2-((1-methyl-1- dodecylpyrrolidin-1-ium-2-ylidene)amino)ethan-1-ol bromide.
